# Lifestyle factors associated with being overweight and obesity in children and adolescents: a cross-sectional study in Zhejiang, China

**DOI:** 10.3389/fpubh.2025.1551099

**Published:** 2025-03-19

**Authors:** Xiaoting Pan, Chaoyi Jiang, Wenqing Wang, Jianfeng Lin

**Affiliations:** The First School of Medicine, School of Information and Engineering, The First Affiliated Hospital of Wenzhou Medical University, Wenzhou Medical University, Wenzhou, China

**Keywords:** lifestyle factors, overweight, obesity, children, adolescents

## Abstract

**Background:**

The prevalence of overweight and obesity among children and adolescents aged 7 to 17 in China is increasingly concerning. Additionally, there is a notable phenomenon where height does not correspond with weight. This study aimed to investigate the prevalence of overweight and obesity in this demographic, explore associations with lifestyle factors, and propose strategies for health promotion.

**Methods:**

This study combines a questionnaire survey with field interviews. Using stratified cluster random sampling, we selected 2,243 primary and middle school students from 49 counties in Zhejiang, China, for the questionnaire survey. Furthermore, we conducted semi-structured interviews with 52 parents.

**Results:**

Among the 2,243 participants surveyed, 523 were classified as either overweight or obese, resulting in a detection rate of 23.32%. The prevalence of overweight and obesity was higher in boys compared to girls (25.6% vs. 21.1%, *p* = 0.01). Additionally, the rates among adolescents and children in urban areas were greater than those in rural areas (25.8% vs. 20.5%, *p* = 0.003). Logistic regression analysis identified several risk factors for overweight and obesity: male gender (OR = 1.846; CI = 1.287 ~ 2.649; *p* = 0.001), 7 days/week the highest frequency of fried food consumption per week (OR = 88.293; CI = 49.369 ~ 157.905; *p* < 0.001), 7 days/week the highest frequency of night snacking (OR = 13.24; CI = 7.176 ~ 24.429; *p* < 0.001), and diets prefer sweetness (OR = 26.299; CI = 15.073 ~ 45.885; *p* < 0.001), saltiness (OR = 14.672; CI = 8.972 ~ 23.992; p < 0.001), and spiciness (OR = 1.967; CI = 1.125 ~ 3.438; *p* = 0.018). Conversely, 7 days/week the highest frequency of moderate-to- vigorous physical activities per week was associated with a lower risk of overweight and obesity (OR = 0.137; CI = 0.057 ~ 0.328; *p* < 0.001). Notably, 84% of parents reported that their children rarely shared interesting stories during school breaks, highlighting the common occurrence of “quiet ten minutes” between classes.

**Conclusion:**

The issue of overweight and obesity among children and adolescents in China is significant, particularly among boys in primary schools. An obesogenic environment contributes to this problem, influenced by changes in dietary habits, exercise patterns, and sociocultural factors. Preventing and controlling overweight and obesity among adolescents and children requires the collective efforts from all sectors of society, including government departments, educational institutions, communities and families. Preventative measures should include lifestyle modifications including exercise and dietary adjustments.

## 1 Introduction

Overweight and obesity in children and adolescents have emerged as a significant global public health issue, with lasting health implications throughout the life course (1, 2). According to the World Health Organization (WHO), the prevalence of obesity among this demographic has increased markedly over the past few decades (1, 3). In 2022, more than 390 million children and adolescents aged 5 to 19 were classified as overweight, including 160 million who were living with obesity. The prevalence of overweight and obesity in this age group surged from just 8% in 1990 to 20% in 2022. In China, the situation mirrors this global trend, with the rate of overweight and obesity among children and adolescents rising from 5.7% in 1992 to 19% in 2022. This means that approximately one in five children and adolescents in China now have a high body mass index (BMI) (4). BMI system is used internationally and is calculated according to the formula (weight/height^2^) (5). Overweight is BMI-for-age greater than 1 standard deviation above the WHO Growth Reference median and obesity is greater than 2 standard deviations above the WHO Growth Reference median (6). The “Healthy China 2030” plan emphasizes the urgent need for interventions to address overweight and obesity among children and adolescents.

Research indicates that children with obesity are more likely to remain obese into adulthood compared to their peers. They also face a higher risk of developing various non-communicable diseases, including endocrine disorders, type 2 diabetes, sleep disorders, and cardiovascular diseases (7, 8). Additionally, being overweight or obese during childhood negatively affects mental health and social adaptation, with these issues often persisting into adulthood (9).

Since the 1990s, China has experienced significant social and economic changes, which have been accompanied by shifts in lifestyle. These changes encompass various aspects, including living and working environments, dietary patterns, and food availability (10, 11). The recent increase in overweight and obesity among children and adolescents may be linked to genetic, demographic, environmental, and lifestyle factors (12, 13). This study aimed to explore the factors affecting overweight and obesity in this population by examining their dietary behaviors, exercise habits, self-awareness, and family circumstances, thereby providing a scientific basis for effective intervention measures.

## 2 Materials and methods

### 2.1 Study design and participants

A school-based, cross-sectional survey was conducted across 7 elementary schools and secondary schools in the Zhejiang province of China during July–October 2023. Stratified cluster sampling was performed to select the study samples. Four elementary schools and three secondary schools were selected in the urban areas (i.e., Hangzhou, Ningbo, Wenzhou, Shaoxing). One elementary school and two secondary schools were selected in the rural areas (i.e., Lishui, Jinhua, and Jiaxing). For each grade, we selected approximately 40 students from these 7 schools. A total of 2,712 students aged 7–17 years were finally included in this study and asked to complete a questionnaire along with their parents at their respective homes. Based on the deletion of extreme values, 2,243 questionnaires were considered valid, with an effectiveness rate of 82.7%.

Further, semi-structured interviews were held with 52 parents of overweight and obese adolescents and children, as mentioned in the questionnaires. The mean interview duration was 32.1 min.

### 2.2 Study question/survey measures

The majority of the study questions were adapted from the 2019 National Student Physical Health Research, wherein most of the key questions were tested for their reliability and validity. The questionnaire collected information based on demographics, physical activities, and dietary behaviors. Semi-structured interviews conducted along with the participants’ parents were about their children’s exercise habits and dietary behaviors, but more in-depth.

### 2.3 Anthropometric measures

Self-reported weight and height were collected to calculate the BMI for each participant. BMI [expressed in weight (kg)/height^2^ (m^2^)] is currently internationally recognized to classify children’s weight status as “non-overweight” and “overweight” (including obese), although the criteria vary across ages and genders. In November 2023, the Institute of Child and Adolescent Health at Peking University issued the “Classification Standards for BMI Overweight and Obesity Screening of Chinese School-aged Children and Adolescents,” which refers to age- and sex-specific BMI. This survey employed Chinese standards and age- and gender-specifics to identify overweight and obese children and adolescents. In this study, the concepts of being overweight and obesity were combined to differentiate children and adolescents with weights within the standard range.

### 2.4 Dietary habits

Information about the respondents’ dietary habits was collected by using several questions, including those on dietary patterns, eating velocity, and taste preferences. Parents along with their children were asked to input the child’s daily consumption of fruits, vegetables, beans, fried food, and midnight snacks. The frequencies of these dietary patterns were classified as “Hardly eat,” “eat 1–3 times/week,” “eat 4–6 times/week,” and “eat 7 times/week.”

### 2.5 Physical activity

Regarding the extent of physical activity, at least 60 min of moderate-to-vigorous-intensity physical activity every day was considered to be sufficient. The subjects were asked to measure their intensity of physical activity. The frequencies of physical activity were classified as “Hardly exercise,” “exercise 1–3 times/week,” “exercise 4–6 times/week,” and “exercise 7 times/week.”

### 2.6 Statistical analysis

Statistical analysis for the questionnaire was performed with reference to the SPSS version 22 (SPSS Inc., Chicago, IL). We used chi-squared analysis to examine the differences in the prevalence of overweight and obesity. Logistic regression analysis was conducted to examine the association between lifestyle factors and BMI classification. Statistical significance was set at *p* < 0.05.

## 3 Results

### 3.1 Characteristics of study participants

Table 1 presents the socio-demographic characteristics of the study participants. Among the 2,243 individuals surveyed, 523 were classified as either overweight or obese, resulting in a detection rate of 23.32%. The prevalence of overweight and obesity was higher in boys compared to girls (25.6% vs. 21.1%, *p* = 0.01). Additionally, the rates of overweight and obesity among adolescents and children in urban areas were greater than those in rural areas (25.8% vs. 20.5%, *p* = 0.003). Variations in the prevalence of overweight and obesity were also observed across the four age categories and three family income categories, as illustrated in Table 1.

**Table 1:** Sociodemographic correlates of overweight and obesity in Chinese children and adolescents.

Variables	Total (*n* = 2,243)	Overweight/obesity		*χ*^2^test	*p*-value
		*n*	%		
Sex					
Boy	1,108	284	25.6	6.562	0.010
Girl	1,135	239	21.1		
Learning period					
Grade 1–3 of primary school	265	63	23.8	12.203	0.007
Grade 4–6 of primary school	518	148	28.6		
Junior high school	839	171	20.4		
Senior high school	621	141	22.7		
Living area					
Urban	1,189	307	25.8	8.866	0.003
Rural	1,054	216	20.5		
Family income(Yuan/m)					
≤5,000	680	117	17.2	20.814	<0.001
<10,000	687	173	25.2		
≥10,000	876	233	26.6		

### 3.2 Factors associated with overweight/obesity

Table 2 outlines the dietary habits related to overweight and obesity. The chi-square test revealed statistically significant associations between the frequency of consuming fried foods, fresh vegetables, and late-night snacks and the prevalence of overweight and obesity (*p* < 0.001). Additionally, a significant relationship was found between food taste and overweight/obesity (p < 0.001). However, no significant association was observed between eating speed and overweight/obesity (*p* = 0.198).

**Table 2:** Associations between overweight, obesity, and dietary habits.

Variables		Total (*n* = 2,243)	Overweight/obesity		*χ*^2^test	*p*-value
			*n*	**%**		
Fried food (d/wk)	0	862	50	5.8	638.407	<0.001
	1–3	519	98	18.9		
	4–6	499	113	22.6		
	7	363	262	72.2		
Vegetables (d/wk)	0	388	241	62.1	465.345	<0.001
	1–3	590	135	22.9		
	4–6	395	91	23.0		
	7	870	56	6.4		
Fruits (d/wk)	0	458	103	22.5	6.625	0.085
	1–3	595	137	23.0		
	4–6	665	140	21.1		
	7	525	143	27.2		
Soy and soy product (d/wk)	0	428	84	19.6	6.832	0.077
	1–3	678	178	26.3		
	4–6	737	166	22.5		
	7	400	95	23.8		
Milk (d/wk)	0	374	79	21.1	106.420	<0.001
	1–3	513	100	19.5		
	4–6	832	136	16.3		
	7	524	208	39.7		
Late night snacks (d/wk)	0	539	44	8.2	291.150	<0.001
	1–3	691	100	14.5		
	4–6	560	150	26.8		
	7	453	229	50.6		
Eating speed (min)	≤10	494	129	26.1	4.669	0.198
	<20	858	201	23.4		
	<30	579	119	20.6		
	≥30	312	74	23.7		
Food taste	Light	757	80	10.6	182.497	<0.001
	Salty	598	178	29.8		
	Spicy	376	57	15.2		
	Sweet	512	208	40.6		

Table 3 outlines the physical activity patterns related to overweight and obesity. The chi-square test indicated a significant association between the frequency and intensity of exercise and weight status (*p* < 0.001).

**Table 3:** Associations between overweight, obesity, and physical activities.

Variables		Total (*n* = 2,243)	Overweight/obesity		*χ*^2^test	*p*-value
			*n*	%		
Moderate-to-vigorous PA ≥ 60 min/d (d/wk)	0	324	190	58.6	268.484	<0.001
	1–3	914	164	17.9		
	4–6	811	126	15.5		
	7	194	43	22.2		

The results of the logistic regression analysis, which examined the relationships among physical activity patterns, dietary habits, and overweight/obesity, are presented in Table 4. The findings revealed that factors such as gender, family income, frequency of consuming fried foods, fresh vegetables, late-night snacks, dietary preferences, and both the frequency and intensity of exercise significantly influence the weight status of adolescents and children. Specifically, male students (OR = 1.846), high weekly consumption of fried foods (OR = 88.293), frequent late-night snacking (OR = 13.24), and a preference for sweet (OR = 26.299), salty (OR = 14.672), and spicy flavors (OR = 1.967) were identified as risk factors for overweight and obesity. Conversely, a high frequency of effective exercise days per week was associated with a reduced risk of overweight and obesity (OR = 0.137).

**Table 4:** Factors associated with overweight and obesity on logistic regression analysis.

Variables	*χ*^2^test	*p*-value	OR (95%CI)
Sex			
Girl			1.000
Boy	11.092	0.001	1.846 (1.287 ~ 2.649)
Learning period			
Grade 1–3 of primary school			1.000
Grade 4–6 of primary school	1.522	0.217	1.465 (0.799 ~ 2.686)
Junior high school	0.021	0.885	0.959 (0.540 ~ 1.703)
Senior high school	0.024	0.877	1.048 (0.579 ~ 1.899)
Living area			
Rural			1.000
Urban	22.710	<0.001	0.171 (0.083 ~ 0.354)
Family income (Yuan/m)			
≤5,000			1.000
<10,000	4.549	0.033	1.669 (1.042 ~ 2.673)
≥10,000	19.946	<0.001	2.632 (1.721 ~ 4.025)
Fried food (d/wk)			
0			1.000
1–3	25.839	<0.001	3.614 (2.202 ~ 5.931)
4–6	36.289	<0.001	4.705 (2.843 ~ 7.787)
7	228.208	<0.001	88.293 (49.369 ~ 157.905)
Vegetables (d/wk)			
0			1.000
1–3	37.466	<0.001	0.217 (0.133 ~ 0.354)
4–6	57.404	<0.001	0.135 (0.081 ~ 0.227)
7	190.630	<0.001	0.019 (0.011 ~ 0.033)
Milk (d/wk)			
0			1.000
1–3	0.198	0.657	1.140 (0.639 ~ 2.035)
4–6	2.456	0.117	1.526 (0.899 ~ 2.590)
7	20.408	<0.001	3.445 (2.014 ~ 5.892)
Late night snacks (d/wk)			
0			1.000
1–3	16.516	<0.001	3.549 (1.927 ~ 6.537)
4–6	6.205	0.013	2.160 (1.178 ~ 3.959)
7	68.232	<0.001	13.240 (7.176 ~ 24.429)
Food taste			
Light			1.000
Salty	114.581	<0.001	14.672 (8.972 ~ 23.992)
Spicy	5.632	0.018	1.967 (1.125 ~ 3.438)
Sweet	132.542	<0.001	26.299 (15.073 ~ 45.885)
Moderate-to-vigorous PA ≥ 60 min/d (d/wk)			
0			1.000
1–3	65.136	<0.001	0.117 (0.069 ~ 0.197)
4–6	56.52	<0.001	0.049 (0.023 ~ 0.108)
7	19.910	<0.001	0.137 (0.057 ~ 0.328)

## 4 Discussion

The increasing prevalence of overweight and obesity among Chinese children and adolescents is a significant public health concern. The findings of this study indicate that the rates of overweight and obesity in this demographic have reached a critical level, with an overall prevalence of 23.32% (25.6% in boys and 21.1% in girls). This aligns with other research conducted in China (14). However, the current rates of overweight and obesity among Chinese children and adolescents are several percentage points lower than those reported in some developed countries, such as the United States (35.5% in boys and 34.1% in girls), New Zealand (29.2% for both boys and girls), and Australia (25.8% in boys and 24.0% in girls) (15).

Our research data also shows the current rates of Chinese children and adolescents’ overweight and obesity are lower than some European countries (Greece 37.3%, Italy 36.8%, Malta 36.7%, Andorra 35.8%, Israel 35%, Spain 34.1%, Cyprus 33.1%, Portugal 32.4%, UK 31.1%, Ireland 31%, France 30%) (16). Childhood and adolescents obesity is also a growing public health problem in other Asian countries. In Korea, the prevalence of obesity among adolescents was 5.9% in 2006 but increased to 11.1% in 2019 (17). A decreasing trend in childhood with a POW-30 (Data on the percentage of overweight ≥30%) was observed from 2009 to 2011 in Japan (18).

The study aimed to provide recent estimates of the prevalence of overweight and obesity among Chinese children and adolescents, as well as to explore the associations between lifestyle habits and weight excess in this population. A previous study has indicated that obesity in school-aged children is linked to inadequate physical activity, excessive sedentary behavior, and poor dietary habits. Our findings further support this by demonstrating that factors such as suboptimal physical activity, unhealthy eating habits, low self-awareness of obesity, and varying family circumstances are associated with obesity (19–21). Understanding the different factors affecting overweight and obesity among school-aged students is crucial for developing effective public policies aimed at reducing these conditions.

### 4.1 Analysis of the characteristics of lifestyle patterns of children and adolescents

In recent decades, China has experienced significant transformations in economic growth, social and cultural dynamics, food and living environments, and technological advancements, all of which have profoundly altered the lifestyle patterns of children and adolescents.

Today, the Internet plays an increasingly central role in the lives of Chinese children and adolescents, offering convenience while also becoming a major factor affecting their health (22). The widespread use of the Internet provides adolescents with access to new ideas and knowledge, enhances opportunities for social support, and increases their chances of obtaining health promotion information. Conversely, excessive use of the Internet and digital media can have detrimental effects on outdoor activities, sleep, and learning. This overuse is associated with an increased risk of weight gain and depression, which negatively impacts healthy lifestyles (23, 24). Additionally, Chinese students often face overwhelming academic pressures that consume much of their time for extracurricular activities (25). Furthermore, physical education programs tend to prioritize safety, which can limit the effectiveness of fostering students’ exercise habits and motivation to engage in physical activity (26).

According to the China Health and Nutrition Survey (CHNS), Chinese children and adolescents are increasingly drawn to high-energy, low-nutrition foods. At the same time, a growing number of advertisements promote appealing but high-calorie foods, which significantly influences the consumption behaviors of children and adolescents.

### 4.2 Relationship between dietary habits and weight status

Previous studies have shown that unhealthy dietary habits are linked to overweight and obesity (27, 28). Our findings indicate that a high consumption of fried foods, late-night snacks, and sugary items is associated with an increased risk of overweight and obesity. Conversely, a higher frequency of consuming fresh vegetables is correlated with a lower risk of these conditions. These results are consistent with the work of Duncan JS, Eng S (29, 30).

Over the past 20 to 30 years, China has undergone significant changes in dietary patterns. According to the food frequency questionnaire (FFQ) used in China, dietary habits can be primarily categorized into traditional Chinese dietary patterns and Westernized dietary patterns. Traditional Chinese dietary patterns are characterized by a high intake of beans, grains, fresh vegetables, and tubers. In contrast, westernized dietary patterns are marked by the consumption of high-energy and high-sugar foods, including cakes, sweets, fast foods, and sugary beverages. The rapid economic development, along with the continuous increase in the production and availability of high-energy foods and the swift expansion of the Western fast-food industry, are considered key factors contributing to an obesogenic environment. A survey indicates that over 90% of urban children and adolescents in China have consumed Western fast food (31). Urban teenagers and children who eat Western fast food frequently (4–5 times a month) have a 1.7% higher incidence of obesity compared to those who consume it less often (1–2 times a month) (32). To further investigate the impact of dietary habits on obesity, we interviewed 52 parents of the questionnaire respondents. Among these parents, 8 reported that their children attend private schools that provide late-night snacks, primarily consisting of Western fast foods such as French fries and hamburgers. The interviewed parents expressed concerns that these late-night snacks, particularly the Western fast food offered at school, contribute to their children’s weight gain.

### 4.3 Relationship between physical activity and weight status

Physical activity is essential for healthy development and is recommended as an effective means to reduce the prevalence of overweight and obesity (33–35), thereby lowering associated health risks among children and adolescents (36, 37). Our studies indicate that physical inactivity is linked to weight gain. The World Health Organization (WHO) guidelines recommend that children and adolescents aged 5 to 17 engage in at least 60 min of moderate-to-vigorous physical activity (MVPA) each day. This can include play, sports, transportation, and structured exercise or training sessions (38, 39). Additionally, it is suggested that schools incorporate at least 30 min of daily exercise into their routines. The “Healthy China 2030” Plan Outline also emphasizes the importance of promoting a healthy lifestyle among students. Despite these well-documented benefits and established guidelines, physical activity levels among Chinese school students remain low (40). According to the Global Report on Physical Activity of Children and Adolescents released in 2018, Chinese children and adolescents ranked last among the 49 countries surveyed in terms of physical activity levels. The 2016 National Survey on the Effectiveness of Physical Fitness for Primary and Secondary School Students revealed that only 29.9% of the 116 million children surveyed met the recommended guideline of engaging in “at least 60 min of moderate to vigorous exercise per day.” In our study, which included 2,243 primary and secondary school students, only 22.2% reported achieving a daily physical activity level of 60 min or more. Based on the World Health Organization’s recommendation for moderate-to-vigorous-intensity physical activity (MVPA) of at least 60 min/day, approximately 87.9% of respondents were classified as insufficiently active.

A survey of 1,908 parents of primary and secondary school students revealed that 75.2% of respondents noted the common occurrence of “quiet ten-minute breaks between classes,” with this phenomenon being more prevalent in primary schools (77.2%) compared to junior high schools (69.8%) (41). Among the 52 parents interviewed, 42 reported that their children rarely discuss interesting events that happen during school breaks, and many mentioned that “for safety reasons, children are not allowed to play on the playground during breaks.” Additionally, the rise in private car usage has led most parents to drive their children to and from school, replacing walking and cycling. Collectively, these factors contribute to reduced energy expenditure and insufficient physical activity among adolescents and children.

Sedentary behaviors are also considered one of the important factors leading to weight gain in adolescents and children. With the rapid popularization of the internet, electronic devices, as well as mobile phones, screen-based activities have become the most common sedentary activities among teenagers and children (42). A systematic review conducted in China in 2020, involving 17 studies and a total of 53,489 children, showed a positive correlation between screen-based sedentary behaviors and an increase in obesity among school-age children in China (43).

### 4.4 Relationship between socioeconomic and sociocultural factors and weight status

Sociocultural factors play a significant role in the occurrence and development of overweight and obesity among adolescents and children. In traditional Chinese culture, being overweight is often viewed as a symbol of health and good fortune. Many people, especially from older generations, believe that chubby children are healthy. Historically, due to material scarcity, traditional beliefs held that being overweight was a sign of family wealth and physical well-being. As a result of these cultural influences, many parents encourage their children to eat as much as possible rather than promoting moderation; the quantity of food consumed often becomes a measure of their child’s health. Unconsciously shaped by these traditional beliefs, many children do not have an objective perception of their weight. Among the 523 overweight and obese children and adolescents surveyed, 57.6% believed their weight was normal, 9.2% thought they were underweight, and only 33.2% recognized that they were overweight. Research has shown that health consciousness can help children and adolescents develop positive habits and maintain a healthy lifestyle (44, 45). On the other hand, low health awareness may lead to a range of health-risk behaviors such as insufficient physical activity and poor dietary choices, ultimately increasing the risk of overweight and obesity (46).

With the rapid development of the economy, the pace of society is getting faster, and social pressure is increasing. More women are going out to work and spending less time with children, which to some extent affects family dietary habits. More families choose to eat outside. The increase in the frequency of dining out makes it easier for children and adolescents to be exposed to unhealthy eating environments, resulting in nutritional and energy imbalances, which can lead to the occurrence of obesity. Similar study also refers that inappropriate diet, a shortage of fresh food in the diet, and consumption of unhealthy food are related to weight status (5).

## 5 Conclusion

Preventing and controlling overweight and obesity among adolescents and children requires the collective efforts from all sectors of society. Government departments should take a dominant position in the prevention system and propose effective obesity prevention policies to create a supportive social environment. With the support of the government, school-family-community cooperative education is encouraged for better management of body weight and promotion of healthy growth and development in children and adolescents. In terms of physical activities, schools should explore sports teaching and activity forms suitable for different age groups and guide students to carry out physical exercise. What’s more, offering one physical education class every day is an integral part of ensuring students to take at least 1 h a day of moderate or above intensity physical activity. The community can provide more diverse sport facilities, and parents should limit screen time for electronic devices and accompany their children to participate in outdoor physical exercise. Diets is also an unignorable problem. Schools and parents need to provide fresh fruits and vegetables to ensure the intake of nutrients. It is also important to create a good dining atmosphere and cultivate children’s habit of focusing on eating. The community can regularly carry out nutrition popularization lectures to pass on scientific health knowledge and nutrition concepts to children and families. Under the leadership of the government, school-family-community system will work effectively to solve new situations and new problems faced by students in their growth.

## 6 Limitations of study

Due to the availability and comparability of data, this study was a cross-sectional design and only investigated some aspects in a specific period of time, without conducting longitudinal dynamic analysis, so it may not reveal the dynamic change trend of overweight and obesity of children and adolescents. Secondly, BMI is a relatively simple index for evaluating overweight and obesity. In the future, we can further increase the measurement of body fat percentage to evaluate obesity in children and adolescents more comprehensively. Finally, this study was only conducted in Zhejiang Province. Due to the obvious regional differences in China and the fact that obesity may also be related to regional characteristics such as eating habits, customs and cultures in different places, the extrapolation of the conclusions of this study should be used with caution.

## Data Availability

The raw data supporting the conclusions of this article will be made available by the authors, without undue reservation.

## References

[1] Zhang X Liu J Ni Y Yi C Fang Y Ning Q. Global prevalence of overweight and obesity in children and adolescents: a systematic review and Meta-analysis. JAMA Pediatr. (2024) 178:800–13. doi: 10.1001/jamapediatrics.2024.1576, PMID: 38856986 PMC11165417

[2] Liu D Zhao LY Yu DM Ju LH Zhang J Wang JZ. Dietary patterns and association with obesity of children aged 6^−^17 years in medium and small cities in China: findings from the CNHS 2010^−^2012. Nutrients. (2018) 11. doi: 10.3390/nu11010003, PMID: 30577428 PMC6356437

[3] NCD Risk Factor Collaboration. Worldwide trends in underweight and obesity from 1990 to 2022: a pooled analysis of 3663 population-representative studies with 222 million children, adolescents, and adults. Lancet. (2021) 403:1027–50. doi: 10.1016/s0140-6736(23)02750-2, PMID: 38432237 PMC7615769

[4] Society CN. Chinese dietary guidelines 2022. Beijing: People's Medical Publishing House (2022). 360 p.

[5] Nittari G Tomassoni D Di Canio M Traini E Pirillo I Minciacchi A. Overweight among seafarers working on board merchant ships. BMC Public Health. (2019) 19:45. doi: 10.1186/s12889-018-6377-6, PMID: 30626365 PMC6327391

[6] de Onis M Onyango AW Borghi E Siyam A Nishida C Siekmann J. Development of a WHO growth reference for school-aged children and adolescents. Bull World Health Organ. (2007) 85:660–7. doi: 10.2471/blt.07.043497, PMID: 18026621 PMC2636412

[7] Steinl D Holzerny P Ruckdäschel S Fäh D Pataky Z Peterli R. Cost of overweight, obesity, and related complications in Switzerland 2021. Front Public Health. (2024) 12:1335115. doi: 10.3389/fpubh.2024.1335115, PMID: 39071145 PMC11282501

[8] GBD 2019 Risk Factors Collaborators. Global burden of 87 risk factors in 204 countries and territories, 1990-2019: a systematic analysis for the global burden of disease study 2019. Lancet. (2020) 396:1223–49. doi: 10.1016/s0140-6736(20)30752-2, PMID: 33069327 PMC7566194

[9] Jebeile H Kelly AS O'Malley G Baur LA. Obesity in children and adolescents: epidemiology, causes, assessment, and management. Lancet Diabetes Endocrinol. (2022) 10:351–65. doi: 10.1016/s2213-8587(22)00047-x, PMID: 35248172 PMC9831747

[10] Wu Y Xue H Wang H Su C Du S Wang Y. The impact of urbanization on the community food environment in China. Asia Pac J Clin Nutr. (2017) 26:504–13. doi: 10.6133/apjcn.032016.09, PMID: 28429917

[11] Wang Y Zhao L Gao L Pan A Xue H. Health policy and public health implications of obesity in China. Lancet Diabetes Endocrinol. (2021) 9:446–61. doi: 10.1016/s2213-8587(21)00118-2, PMID: 34097869

[12] Alhowikan AM Alsharqawy NE Alazmaa NN Saeed AI Alhazzani YA Alhowaish NY. Lifestyle habits and obesity indices among male adolescents in Riyadh, Saudi Arabia. Sci Rep. (2023) 13:12099. doi: 10.1038/s41598-023-37920-5, PMID: 37495635 PMC10372041

[13] Kerkadi A Sadig AH Bawadi H Al Thani AAM Al Chetachi W Akram H. The relationship between lifestyle factors and obesity indices among adolescents in Qatar. Int J Environ Res Public Health. (2019) 16. doi: 10.3390/ijerph16224428, PMID: 31766192 PMC6888352

[14] Zhang Y Lou H Huang Y Wang R Wen X Wu C. Trends of overweight and obesity prevalence in school-aged children among Henan Province from 2000 to 2019. Front Public Health. (2022) 10:1046026. doi: 10.3389/fpubh.2022.1046026, PMID: 36544796 PMC9760942

[15] Duncan S Duncan EK Fernandes RA Buonani C Bastos KD Segatto AF. Modifiable risk factors for overweight and obesity in children and adolescents from São Paulo. Brazil BMC Public Health. (2011) 11:585. doi: 10.1186/1471-2458-11-585, PMID: 21781313 PMC3154175

[16] Nittari G Scuri S Petrelli F Pirillo I di Luca NM Grappasonni I. Fighting obesity in children from European World Health Organization member states. Epidemiological data, medical-social aspects, and prevention programs. Clin Ter. (2019) 170:e223–30. doi: 10.7417/ct.2019.2137, PMID: 31173054

[17] Lee S Lee HJ Lee KG Kim J. Obesity and high myopia in children and adolescents: Korea National Health and nutrition examination survey. PLoS One. (2022) 17:e0265317. doi: 10.1371/journal.pone.0265317, PMID: 35333875 PMC8956184

[18] Tanaka H Morisaki N Piedvache A Harada S Urayama KY. Trends in obesity and blood lipid abnormalities in school-age children in Japan. Pediatr Int. (2021) 63:825–32. doi: 10.1111/ped.14544, PMID: 33188709

[19] O'Malley G Ring-Dimitriou S Nowicka P Vania A Frelut ML Farpour-Lambert N. Physical activity and physical fitness in pediatric obesity: what are the first steps for clinicians? Expert conclusion from the 2016 ECOG workshop. Int J Exerc Sci. (2017) 10:487–96. doi: 10.70252/YFYO2813, PMID: 28674594 PMC5466409

[20] Mahfouz AA Abdelmoneim I Khan MY Daffalla AA Diab MM Al-Gelban KS. Obesity and related behaviors among adolescent school boys in Abha City, southwestern Saudi Arabia. J Trop Pediatr. (2008) 54:120–4. doi: 10.1093/tropej/fmm089, PMID: 18039676

[21] Patrick K Norman GJ Calfas KJ Sallis JF Zabinski MF Rupp J. Diet, physical activity, and sedentary behaviors as risk factors for overweight in adolescence. Arch Pediatr Adolesc Med. (2004) 158:385–90. doi: 10.1001/archpedi.158.4.385, PMID: 15066880

[22] Ma Y Wu H Shen J Wang J Wang J Hou Y. Correlation between lifestyle patterns and overweight and obesity among Chinese adolescents. Front Public Health. (2022) 10:1027565. doi: 10.3389/fpubh.2022.1027565, PMID: 36408045 PMC9670141

[23] Reid Chassiakos YL Radesky J Christakis D Moreno MA Cross C. Children and adolescents and digital media. Pediatrics. (2016):138. doi: 10.1542/peds.2016-2593 27940795

[24] Durmus G Ortabag T Ozdemir S. Determining the relationship between obesity and problematic internet use among adolescents. Iran J Public Health. (2021) 50:1796–804. doi: 10.18502/ijph.v50i9.7052, PMID: 34722375 PMC8542835

[25] Bx L. Studying burden’s increasing mechanism and radical control path in basic schools. Nanjing J Social Sci. (2021):146–55. doi: 10.15937/j.cnki.issn1001-8263.2021.10.018

[26] Peihong LI Mei W. Investigation on Chinese children and youth physical activity status and influence factors. Chinese J School Health. (2016) 37:805-809+813. doi: 10.16835/j.cnki.1000-9817.2016.06.002

[27] Shang XW Liu AL Zhang Q Hu XQ Du SM Ma J. Report on childhood obesity in China (9): sugar-sweetened beverages consumption and obesity. Biomed Environ Sci. (2012) 25:125–32. doi: 10.3967/0895-3988.2012.02.001, PMID: 22998817

[28] Grimes CA Riddell LJ Campbell KJ Nowson CA. Dietary salt intake, sugar-sweetened beverage consumption, and obesity risk. Pediatrics. (2013) 131:14–21. doi: 10.1542/peds.2012-1628, PMID: 23230077

[29] Duncan JS Schofield G Duncan EK Rush EC. Risk factors for excess body fatness in New Zealand children. Asia Pac J Clin Nutr. (2008) 17:138–47. PMID: 18364339

[30] Eng S Wagstaff DA Kranz S. Eating late in the evening is associated with childhood obesity in some age groups but not in all children: the relationship between time of consumption and body weight status in U.S. children. Int J Behav Nutr Phys Act. (2009) 6:27. doi: 10.1186/1479-5868-6-27, PMID: 19460145 PMC2689163

[31] Ma GS Li YP. Analysis of the relationship between the frequency of Western fast food consumption and obesity rate among children and adolescents in four cities in China. ACTA Nutrimenta SINICA. (2004) 26:486–9.

[32] Chen SMGSM. Guidelines for the prevention and control of overweight and obesity among school-age children and adolescents in China. ACTA Nutrimenta SINICA. (2008) 511

[33] Barlow SE. Expert committee recommendations regarding the prevention, assessment, and treatment of child and adolescent overweight and obesity: summary report. Pediatrics. (2007) 120:S164–92. doi: 10.1542/peds.2007-2329C, PMID: 18055651

[34] Kavey RE Allada V Daniels SR Hayman LL McCrindle BW Newburger JW. Cardiovascular risk reduction in high-risk pediatric patients: a scientific statement from the American Heart Association expert panel on population and prevention science; the councils on cardiovascular disease in the Young, epidemiology and prevention, nutrition, physical activity and metabolism, high blood pressure research, cardiovascular nursing, and the kidney in heart disease; and the interdisciplinary working group on quality of care and outcomes research: endorsed by the American Academy of Pediatrics. Circulation. (2006) 114:2710–38. doi: 10.1161/circulationaha.106.179568 17130340

[35] Jerome GJ Fink T Brady T Young DR Dickerson FB Goldsholl S. Physical activity levels and screen time among youth with overweight/obesity using mental health services. Int J Environ Res Public Health. (2022) 19:19. doi: 10.3390/ijerph19042261, PMID: 35206449 PMC8871648

[36] Hills AP Okely AD Baur LA. Addressing childhood obesity through increased physical activity. Nat Rev Endocrinol. (2010) 6:543–9. doi: 10.1038/nrendo.2010.133 20736922

[37] Hills AP King NA Armstrong TP. The contribution of physical activity and sedentary behaviours to the growth and development of children and adolescents: implications for overweight and obesity. Sports Med. (2007) 37:533–45. doi: 10.2165/00007256-200737060-00006, PMID: 17503878

[38] Bull FC Al-Ansari SS Biddle S Borodulin K Buman MP Cardon G. World Health Organization 2020 guidelines on physical activity and sedentary behaviour. Br J Sports Med. (2020) 54:1451–62. doi: 10.1136/bjsports-2020-102955, PMID: 33239350 PMC7719906

[39] McMurray RG Berry DC Schwartz TA Hall EG Neal MN Li S. Relationships of physical activity and sedentary time in obese parent-child dyads: a cross-sectional study. BMC Public Health. (2016) 16:124. doi: 10.1186/s12889-016-2795-5, PMID: 26851940 PMC4744403

[40] Qin G Qin Y Liu B. Association between BMI and health-related physical fitness: a cross-sectional study in Chinese high school students. Front Public Health. (2022) 10:1047501. doi: 10.3389/fpubh.2022.1047501, PMID: 36568802 PMC9773132

[41] HE SM. The phenomenon of "quiet ten minute break" in primary and secondary schools is common. J Moral Educ Primary Secondary. (2019) 78.

[42] Novaković S Milenković S Srećković M Backović D Ignjatović V Capo N. Children's internet use and physical and psychosocial development. Front Public Health. (2023) 11:1163458. doi: 10.3389/fpubh.2023.1163458, PMID: 37361154 PMC10285096

[43] Mo Z Wang H Zhang B Ding G Popkin BM Du S. The effects of physical activity and sedentary behaviors on overweight and obesity among boys may differ from those among girls in China: an open cohort study. J Nutr. (2022) 152:1274–82. doi: 10.1093/jn/nxab446, PMID: 35018425 PMC9071318

[44] Tsai CL Lin YW Hsu HC Lou ML Lane HY Tu CH. Effects of the health-awareness-strengthening lifestyle program in a randomized trial of Young adults with an at-risk mental state. Int J Environ Res Public Health. (2021) 18:18. doi: 10.3390/ijerph18041959, PMID: 33670454 PMC7922885

[45] Liu YW Lv SR. Preliminary study on main theories of individual health behavior change and integration. Chinese J Health Educ. (2018) 34:284–7. doi: 10.16168/j.cnki.issn.1002-9982.2018.03.023

[46] Kenney EL Gortmaker SL. United States Adolescents' Television, computer, videogame, smartphone, and tablet use: associations with sugary drinks, sleep, physical activity, and obesity. J Pediatr. (2017) 182:144–9. doi: 10.1016/j.jpeds.2016.11.015, PMID: 27988020

